# PTEN stabilizes TOP2A and regulates the DNA decatenation

**DOI:** 10.1038/srep17873

**Published:** 2015-12-10

**Authors:** Xi Kang, Chang Song, Xiao Du, Cong Zhang, Yu Liu, Ling Liang, Jinxue He, Kristy Lamb, Wen H. Shen, Yuxin Yin

**Affiliations:** 1 Institute of Systems Biomedicine, Beijing Key Laboratory of Tumor Systems Biology, Department of Pathology, School of Basic Medical Sciences, Peking University Health Science Center, Beijing 100191, China; 2 Department of Radiation Oncology, Weill Medical College of Cornell University, New York, NY 10065, USA; 3 Peking-Tsinghua Center for Life Sciences, Beijing, 100191, China

## Abstract

PTEN is a powerful tumor suppressor that antagonizes the cytoplasmic PI3K-AKT pathway and suppresses cellular proliferation. PTEN also plays a role in the maintenance of genomic stability in the nucleus. Here we report that PTEN facilitates DNA decatenation and controls a decatenation checkpoint. Catenations of DNA formed during replication are decatenated by DNA topoisomerase II (TOP2), and this process is actively monitored by a decatenation checkpoint in G2 phase. We found that PTEN deficient cells form ultra-fine bridges (UFBs) during anaphase and these bridges are generated as a result of insufficient decatenation. We show that PTEN is physically associated with a decatenation enzyme TOP2A and that PTEN influences its stability through OTUD3 deubiquitinase. In the presence of PTEN, ubiquitination of TOP2A is inhibited by OTUD3. Deletion or deficiency of PTEN leads to down regulation of TOP2A, dysfunction of the decatenation checkpoint and incomplete DNA decatenation in G2 and M phases. We propose that PTEN controls DNA decatenation to maintain genomic stability and integrity.

*PTEN* is one of the most frequently mutated genes in human tumors such as glioblastoma, breast cancer, prostate cancer, endometrial cancer, colon cancer, and lung cancer^1^,^2^,^3^,^4^. Germline mutations of *PTEN* are also found in high cancer susceptibility syndromes such as Cowden Syndrome^5^,^6^. Homozygous deletion of PTEN in mice is embryonically lethal and heterozygous deletion results in spontaneous tumor formation^5^,^7^,^8^,^9^. Complete deletion of PTEN is found in glioblastoma and endometrial cancer and is associated with tumorigenesis in affected tissues^10^,^11^. Recent data from our laboratory show that c-terminal PTEN deletion in mice leads to genomic instability and spontaneous formation of various tumors, including cancers and B cell lymphoma^12^.

The protein encoded by *PTEN* has both lipid and protein phosphatase activity^6^,^13^,^14^. PTEN dephosphorylates phosphoinositide-3,4,5-triphosphate (PIP3), which is an activator of AKT^6^,^13^. Loss of PTEN activates the PI3K-AKT pathway and promotes cell proliferation^14^,^15^. In addition to its canonical tumor suppressor functions in the cytoplasm, there is increasingly abundant evidence that nuclear PTEN is also functions in tumor suppression^16^,^17^,^18^,^19^,^20^,^21^. Nuclear localization of PTEN is essential for suppression of multiple types of tumors, including leukemia, pancreatic tumors, melanoma and colorectal cancer. Absence of nuclear PTEN is strongly associated with a high rate of tumorigenesis and poor prognosis^16^,^17^,^18^,^19^,^20^,^21^.

Before and during mitosis, replicated sister chromatids must be properly decatenated in preparation for anaphase chromosome segregation. Decatenation deficiencies in cancer cells may result in additional chromosome imbalances that increase tumor malignancy^22^. Decatenation of entangled DNA is accomplished by a series of enzymatic reactions catalyzed by DNA topoisomerase II (TOP2)^23^. This post-replication process is monitored by a DNA decatenation checkpoint in G2 phase^21^,^22^,^23^,^24^,^25^. Insufficient resolution of replication generated DNA entanglements activates this checkpoint and delays entrance of cells into mitosis^22^,^24^. This decatenation checkpoint can be activated by catalytic inhibitors of TOP2, such as the bis-(2, 6-dioxopiperazine) derivatives ICRF-193 and ICRF-187, which bind TOP2 and force it into a closed conformation which cannot decatenate DNA^22^,^24^. Attenuation of the decatenation checkpoint contributes to chromosome instability in cancer cells^26^.

There are two topoisomerase II isozymes in mammalian cells, TOP2A and TOP2B^27^. TOP2A functions specifically in chromosome untangling and is essential for segregation of sister chromatids before anaphase^26^. It is also required for decatenation checkpoint activation^24^,^25^. When ICRF-193 treatment gives rise to decatenation errors, TOP2A is fixed in a conformation where the phosphorylation of Ser1524 is exposed. This phosphorylation then recruits MDC1 to DNA and activates the checkpoint^25^. Knock down of TOP2A but not TOP2B abolishes the function of this checkpoint when cells are treated with ICRF-193, which allows cells to proceed through mitosis with considerable genomic damage caused by chromosome instability^21^. In addition to its role in decatenation following replication and the activation of the G2 decatenation checkpoint, TOP2A also functions in mitosis to decatenate centromeric DNA after the removal of cohesin^28^,^29^. Depletion or inhibition of TOP2A results in abnormal anaphase PICH coated bridges^29^,^30^. PICH is an SNF2 family helicase, which localizes at anaphase bridges that are generated by pre-mitosis chromatid organizational errors, such as those generated from replication stress and incomplete decatenation^31^,^32^. These bridges, which are often undetectable by conventional DNA dye staining, are called ultra-fine bridges (UFBs)^31^,^32^,^33^,^34^,^35^,^36^. UFBs which are positive for PICH staining can thus be used as an indicator for pre-mitotic chromatid organizational errors^30^,^31^,^32^,^34^.

At the transcriptional level, p53 negatively regulates expression of *TOP2A*^37^,^38^,^39^. TOP2A protein levels exhibit a dynamic pattern during the cell cycle, with a peak in G2-M and a decrease in G1^26^,^40^. It is known that ubiquitin-dependent degradation of TOP2A is mediated by BRCA1^26^,^41^,^42^. However, details of the mechanism by which its stability is regulated by ubiquitination remain elusive. Previously we reported nuclear PTEN is essential for maintaining chromosomal integrity^20^. Here, we demonstrate PTEN plays a role in regulating TOP2A protein stability by promoting OTUD3 catalyzed TOP2A deubiquitination. PTEN thus facilitates the decatenation process by maintaining TOP2A levels, and loss of PTEN results in decatenation errors which lead to genomic instability^21^.

## Results

### Failure of decatenation in PTEN-deficient cells results in formation of ultra-fine bridges in anaphase

**Figure 1:**
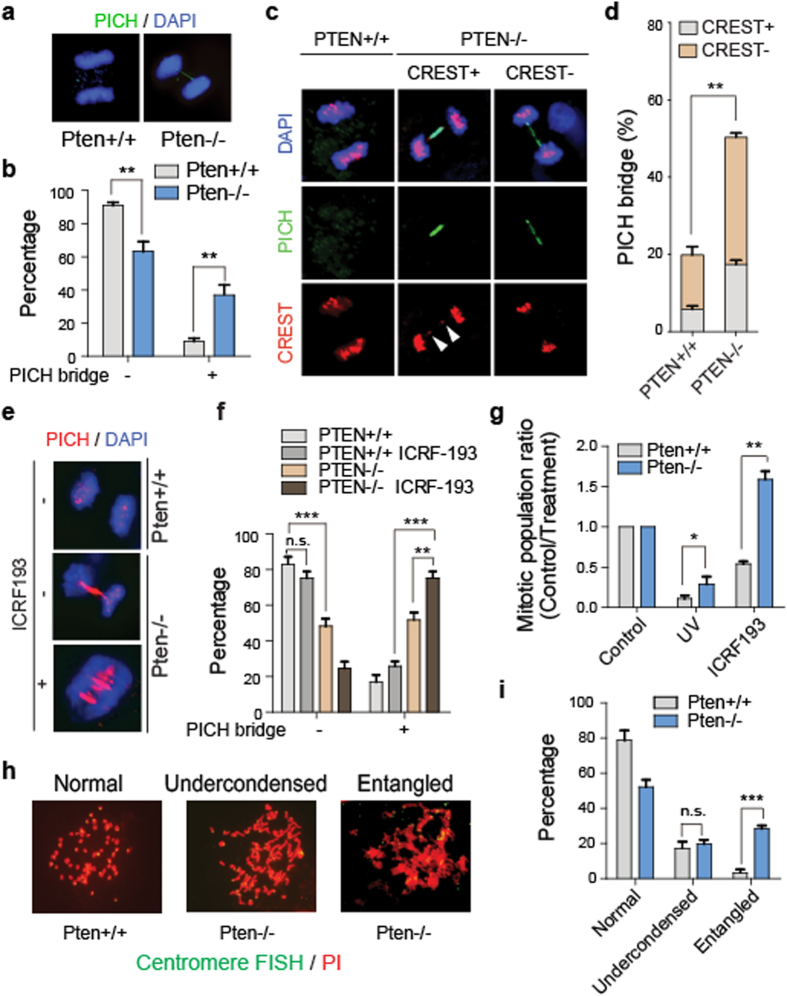
Failure of decatenation in Pten deficient cells results in the formation of ultra-fine bridges in anaphase cells. (**a**) Immunofluorescence of PICH-coated ultra-fine bridges. *Pten*^+/+^ or *Pten*^−/−^ MEFs were stained with the α-PICH antibody (green). Representative anaphase cells are shown with and without PICH-coated bridges. (**b**) Statistical analysis of **a**. Anaphase *Pten*^+/+^ or *Pten*^−/−^ MEFs were counted and grouped based on presence or absence of PICH bridges. Quantification data to determine the percentage of cells with or without PICH bridges are representative of 3 independent experiments ± SEM. n = 40, One-Way ANOVA, **p < 0.01. (**c**) Immunofluorescence of PICH-coated ultra-fine bridges and centromeres. HCT116 *PTEN*^+/+^ and *PTEN*^−/−^ cells were stained for PICH (green), CREST (red) and DNA (DAPI). Cells with no bridges (left); bridges with CREST foci (middle); and bridges without CREST foci (right); (**d**) Statistical analysis of **c**. Stacked column chart illustrating the proportion of cells with PICH bridges positive or negative for CREST staining. Quantification data represent 3 independent experiments ± SEM. n = 40, One-Way ANOVA, **p < 0.01. (**e**) Immunofluorescence of PICH-coated ultra-fine bridges. HCT116 *PTEN*^+/+^ or *PTEN*^−/−^ cells were treated with ICRF-193 prior to PICH (red) immunofluorescent staining. Cells which are illustrated are anaphase cells with no, one or multiple PICH-coated bridges. (**f**) Statistical analysis of **e**. Column chart illustrating the proportion of ICRF-193 treated and untreated HCT116 cells with and without PICH bridges. Quantification data represent 3 independent experiments ± SEM. n = 40, One-Way ANOVA, **p < 0.01, ***p < 0.001. (**g**) Flow cytometry analysis of mitotic cells. MEF cells were treated with UV or ICRF-193 prior to dual-color flow cytometry analysis with phospho-Ser10 histone 3 and PI (propidium iodide) staining. Quantification data represent 3 independent experiments ± SEM. One-Way ANOVA, *p < 0.05, **p < 0.01. (**h**) Metaphase spread followed by centromeric FISH. Shown are representative images of MEF metaphase spreads after 1 μM ICRF-193 treatment for 2 h with normally condensed chromosomes, undercondensed chromosomes, and entangled chromosomes. (**i**) Statistical analysis of (**h**). Column chart showing the proportion of ICRF-193 cells with normal, undercondensed or entangled chromosomes. Quantification data represent 3 independent experiments ± SEM. n = 50, One-Way ANOVA; n.s. no significance, ***p < 0.001.

Chromosome instability (CIN) may resulted from defects in mitotic checkpoints and chromosomal segregation. We demonstrated previously nuclear PTEN maintains chromosomal integrity and contributes to centromeric stability, and loss of PTEN may therefore bring about chromosomal aberrations^12^,^20^,^43^. Indeed, there are more anaphase bridges observed in *Pten*^−/−^ cells as compared with *Pten*^+/+^ cells with DAPI staining (Supplementary Fig. 1a,1b). CIN could be achieved through breakdown of one or multiple maintenance machineries span from S phase to mitosis^26^,^44^. Therefore in order to determine whether PTEN is involved in the pre-mitotic regulation of chromosomal integrity, we stained for UFBs with the SNF2 family helicase PICH^31^,^36^. UFBs are histone-negative bridges, which indicate pre-mitotic chromatid organizational errors. In contrast, DAPI bridges for the most part are generated from mitosis^31^,^32^,^33^,^34^,^35^,^36^. We found that the number of PICH bridges increases in *Pten*^−/−^ cells (Fig. 1a,b), indicating loss of PTEN contributes to errors in pre-mitosis chromatid organization. Previous studies show that UFBs are detected in centromeric regions, providing an evidence that in addition to cohesion of sister chromatid, sister centromeric DNA is also catenated^28^,^29^. We found that approximately 30% of ultra-fine bridges are associated with a lagging centromere by staining with both PICH and CREST antibodies, which reflects a problem with decatenation of centromeric DNA (Fig. 1c,d). This may offer an alternative explanation for the centromeric instability in *PTEN*^−/−^ cells and *Pten* knock-in mice we reported previously^12^,^20^.

UFBs may result from errors in the decatenation process^30^,^31^,^32^,^33^. To determine whether the decatenation process is disrupted in *PTEN*^−/−^ cells, we treated *PTEN*^+/+^ and *PTEN*^−/−^ cells with the decatenation inhibitor ICRF-193. After treatment, fewer mitotic cells were observed by microscopy in *PTEN*^+/+^ cells as compared with *PTEN*^−/−^ cells, indicating a functional G2 decatenation checkpoint prevents cells from entering mitosis. In this mitotic population, an increased number of cells with bridges as well as an increase in the number of bridges per cell were observed (Fig. 1e,f). The percentage of PICH bridges present in *PTEN*^−/−^ anaphase cells showed a greater increase after treatment as compared with *PTEN*^+/+^ cells (Fig. f). These results suggest the differences in the PICH bridge phenotypes of *PTEN*^+/+^ and *PTEN*^−/−^ cells is in part attributable to decatenation.

### Attenuation of the decatenation checkpoint contributes to decatenation aberrations in PTEN-deficient cells

After DNA replication, sister chromatids become entangled. Under normal circumstances, sister chromatid catenation status is monitored by the decatenation checkpoint, which arrests cells in G2 phase until sister chromatids are fully resolved^22^,^24^. As such, the PICH bridges which are identified above (Fig. 1f) most likely result from failure of a decatenation checkpoint. To investigate the role of PTEN in decatenation checkpoint function, we performed flow cytometry with *Pten*^+/+^ and *Pten*^−/−^ MEF cells that had been treated with either UV to activate the DNA damage checkpoint, or with ICRF-193, which inhibits decatenation and activates the decatenation checkpoint. Both of these checkpoints are in G2, and share some but not all of the associated signaling molecules^22^,^25^. As expected, the fraction of *Pten*^+/+^ and *Pten*^−/−^ cells in mitosis was lower in UV-treated cells, and Pten-deficient cells showed more cells in mitosis than the *Pten*^+/+^ cells due to bypass of the DNA damage checkpoint in *Pten*^−/−^ cells^45^ (Fig. 1g, S1c). The fraction of *Pten*^+/+^ cells in mitosis was reduced after treatment with ICRF-193, indicating the decatenation checkpoint in G2 phase prevents cells from proceeding into mitosis. In *Pten*^−/−^ cells, however, the fraction of mitotic cells after ICRF-193 treatment is even greater than the fraction of mitotic cells in the untreated group, suggesting that the decatenation checkpoint has failed to stop cell cycle progression in *Pten*^−/−^ cells (Fig. 1g, S1c). Chromatid entanglements brought about pseudomitosis after ICRF-193 treatment, resulting in accumulation of a mitotic population in *PTEN*^−/−^ cells^21^,^25^ (Fig. 1g, S1c).

To confirm failure of the decatenation checkpoint in Pten-deficient cells, we made centromeric FISH-stained metaphase spreads of ICRF-193 treated *Pten*^+/+^ and *Pten*^−/−^ cells. There was no significant difference in undercondensed chromosomes in *Pten*^+/+^ and *Pten*^−/−^ cells, suggesting these cells have similar sensitivities in their response to ICRF-193. However, there was a significantly larger number of entangled chromosomes in *Pten*^−/−^ cells (Fig. 1h,i). These entangled chromosomes are consistent with the pseudomitosis phenotype, suggesting these cells have entered mitosis in the presence of chromatid entanglements, which is characteristic of decatenation checkpoint failure^21^,^22^,^25^,^46^,^47^.

### PTEN is physically associated with decatenation protein TOP2A

**Figure 2:**
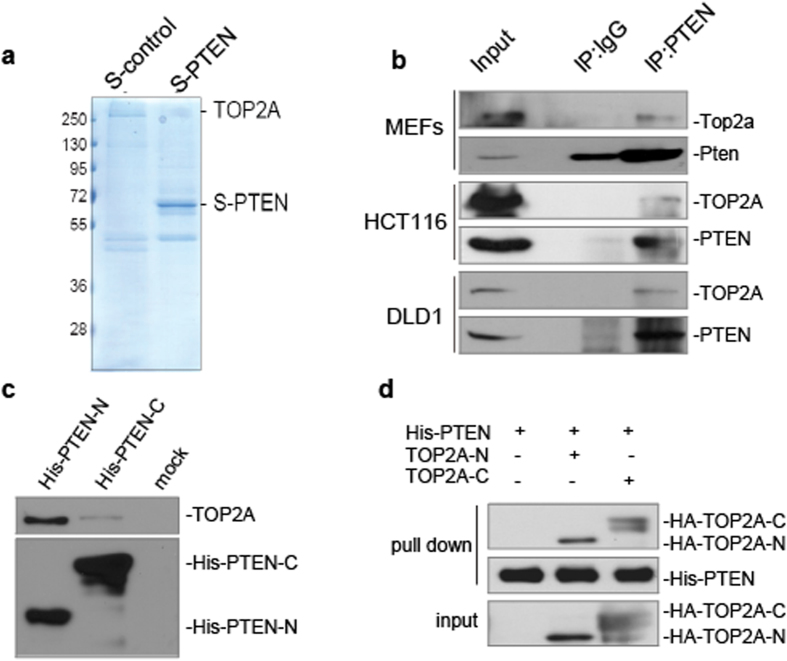
PTEN is physically associated with the critical decatenation protein TOP2A. (**a**) S-tag pull down assays. Coomassie blue-stained gel shows bands sequenced by mass spectrometry. Specific peptides of TOP2A are highlighted (Fig. S2A). (**b**) Co-immunoprecipitation assays. Western blots show TOP2A and PTEN after co-immunoprecipitation with PTEN antibody in MEF, HCT116, and DLD1 cell lysates. (**c**) His pull-down assays. HCT116 cell lysates were incubated with purified His-tagged PTEN C-terminal or N-terminal fragments. Associated TOP2A, His-PTEN-N and His-PTEN-C protein were then evaluated with indicated antibodies. (**d**) *In vitro* binding assays for PTEN and TOP2A interaction. Flag-tagged TOP2A-N or C terminal and His-tagged PTEN were purified and incubated together prior to His-pull down for detection of *in vitro* association. Complex proteins were identified with His and Flag antibodies.

TOP2A plays a critical role in DNA decatenation and decatenation checkpoint activation^21^,^24^,^25^, and this protein was found on our PTEN pull down list (Fig. 2a lane 2, supplementary Fig. 2a), raising the possibility that PTEN may control decatenation partially through TOP2A. Endogenous interaction of these two proteins was confirmed by IP-Western assays in various cell systems, including MEF, HCT116, and DLD1 cells (Fig. 2b lane 3). To identify the site of PTEN which interacts with TOP2A, we performed His-pull down assays using the PTEN N- or C-terminus, and the N-terminus of PTEN showed strong association with TOP2A (Fig. 2c lane 1, 2). This is consistent with the mass spectrometry results from our earlier study of C-terminal deleted Pten mice, in which mutant Pten ΔC acts as gain of function mutant and exhibits dominant negative effects^12^. A weaker physical association of the PTEN C-terminus with TOP2A was also observed, which was not surprising as many nuclear functions of PTEN are C-terminal dependent^12^,^20^,^43^,^48^. To further confirm PTEN and TOP2A interact directly, we performed an *in vitro* pull down assay using purified proteins. We found that full-length PTEN interacts directly with both the N- and C-terminus of TOP2A (Fig. 2d lane 3, 4). In addition, using immunofluorescence and confocal microscopy, we observed co-localization of PTEN and TOP2A in *PTEN*^+/+^ cells (Supplementary Fig. 2b). These data confirm PTEN and TOP2A physically interact.

### PTEN regulates decatenation by maintaining the TOP2A expression level

**Figure 3:**
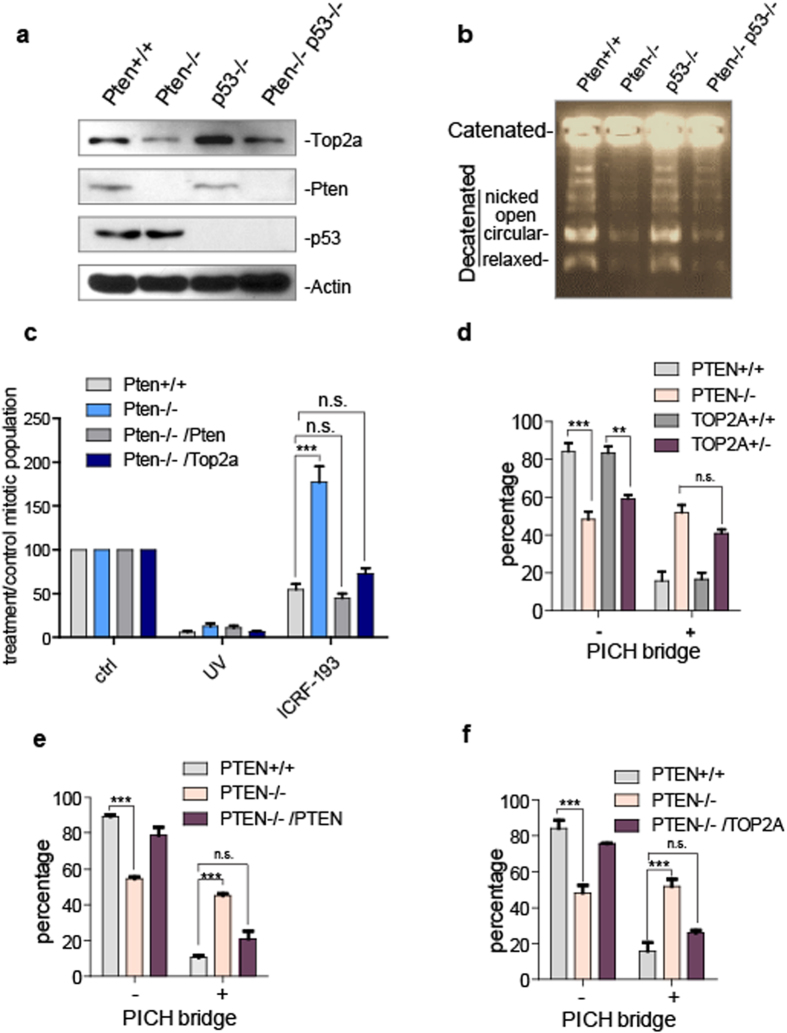
TOP2A decreases in PTEN deficient cells and ectopic expression of PTEN restores TOP2A levels and rescues decatenation deficiencies. (**a**) Western blot analysis. Top2a, Pten and p53 levels in MEF *Pten*^+/+^, MEF *Pten*^−/−^ , MEF *p53*^−/−^ or MEF *Pten*^−/−^*p53*^−/−^ cells were analyzed with western blotting. (**b**) Topoisormerase II activity assays. Nuclear extracts of *Pten*^+/+^, *Pten*^−/−^, *p53*^−/−^, and *Pten*^−/−^ *p53*^−/−^ MEF cells were assayed for the ability to decatenate kinetoplast DNA (kDNA) with 2 ug of total protein added to each reaction system. (**c**) Flow cytometry analysis of mitotic cells. Dual color flow cytometry analysis was performed with phospho-Ser10 histone 3 and PI staining of MEF *Pten*^+/+^ or *Pten*^−/−^ cells. MEF *Pten*^−/−^ cells with restored Pten or Top2a after UV or ICRF-193 treatment. Quantification data represent 3 independent experiments ± SEM, One-Way ANOVA, n.s. no significance, ***p < 0.001. (**d**) Statistical analysis of PICH-bridge positive cells. Anaphase HCT116 *PTEN*^+/+^ or HCT116 *PTEN*^−/−^ cells were counted and grouped based on presence or absence of PICH bridges. Quantification data of percentage of cells with or without PICH bridges represent 3 independent experiments ± SEM. n = 40, One-Way ANOVA, **p < 0.01, ***p < 0.001. (**e**) Statistical analysis of PICH-bridge positive cells. Staining of PICH ultra-fine bridges in HCT116 *PTEN*^+/+^ or *PTEN*^−/−^ cells, and HCT116 *PTEN*^−/−^ cells expressing ectopic PTEN. Quantification data represent 3 independent experiments ± SEM. n = 40, One-Way ANOVA, ***p < 0.001. (**f**) Statistical analysis of PICH bridge positive cells. Column chart illustrating the proportion of cells with and without PICH bridges in HCT116 *PTEN*^+/+^ or *PTEN*^−/−^ cells, and in HCT116 *PTEN*^−/−^ cells expressing ectopic TOP2A. Quantification data represent 3 independent experiments ± SEM. n = 40, One-Way ANOVA, ***p < 0.001.

Based on previous studies, downregulation of TOP2A by knock-down, induced depletion or ubiquitin-mediated degradation is sufficient to give rise to PICH coated UFBs and bring about dysregulation of decatenation^21^,^30^,^42^. To investigate whether the aberrations in decatenation caused by lack of PTEN are due to downregulation of TOP2A, we examined Top2a levels in MEF cells with Pten deletion, p53 deletion or both in the presence of DNase to release chromatin bound protein. In Pten-deficient MEFs, Top2a was expressed at a lower level as compared with *Pten*^+/+^ cells (Fig. 3a lane 1 vs lane 2). p53 is a negative transcriptional regulator of TOP2A^38^,^39^, and it has been reported that loss of PTEN, or partial loss of its C-terminus can activate p53^12^,^49^,^50^. We therefore examined the effects of p53 depletion, and combined Pten/p53 depletion on Top2a levels. We found that the level of Top2a increased in the absence of p53 (Fig. 3a lane 1 vs lane 3) as expected, but decreased again in the absence of both Pten and p53 (Fig. 3a lane 3 vs lane 4). This indicates that regulation of Top2a by Pten is independent of p53 transcriptional regulation. TOP2A is a highly phosphorylated protein^27^, and in order to determine whether PTEN regulates phosphorylation of TOP2A, we employed *in vitro* phosphatase assays followed by mass-spectrometry analysis. We found there were no significant differences in phosphorylation levels of TOP2A in the control group and the PTEN group (Supplementary Form 1).

It has been reported that TOP2A protein levels exhibit a pattern of dynamic change during the cell cycle, with peak levels in G2-M and a decreased levels in G1, while TOP2B protein levels do not fluctuate^26^,^40^. We therefore examined levels of TOP2A in G2-M phase to confirm PTEN regulation occurs when TOP2A is enzymatically active. Cells were synchronized with a double thymidine block before release for 6 h or 8 h. This synchronization resulted in similar enrichment in the proportion of G2/M phase cells, and also resulted in decreases in TOP2A protein levels in both HCT116 *PTEN*^−/−^ cells and MEF *Pten*^−/−^ cells at these two time points. (Supplementary Fig. 3a, 3b lane 1 vs lane 2, lane 3 vs lane 4). In addition, we examined the tissue distribution of Top2a in a CRISPR generated *Pten*^+/−^ mouse model. Top2a showed significant downregulation in the ovary and breast of the *Pten*^+/−^ mouse (Supplementary Fig. 3d lane 1 vs lane 2, lane 7 vs lane 8). As the overall level of TOP2A would be expected to show correlation with the TOP2A catalytic capacity, less activity overall would be expected in these cells. We performed an *in vitro* decatenation assay, using nuclear extracts to decatenate linked DNA substrates. As expected, wild type and p53-deficient MEFs successfully decatenated the substrate to a large extent, while Pten-deficient and Pten/p53-double deficient MEFs were unable to efficiently decatenate the substrate (Fig. 3b lane 1, 3 vs lane 2, 4). These results indicate that PTEN maintains TOP2A protein expression levels, and influences decatenation activity.

### Reintroduction of PTEN or TOP2A restores the decatenation checkpoint and eliminates PICH bridges

To further consolidate our findings regarding downregulation of Top2a expression resulting from Pten deficiency, we transiently transfected exogenous Pten into *Pten*^−/−^ cells and found that the level of Top2a was partially restored (Supplementary Fig. 3c lane 1 vs lane 3). Some nuclear PTEN functions have been shown to be phosphatase activity independent^12^,^18^,^20^,^48^, and to determine whether PTEN regulation of TOP2A is phosphatase dependent, we transfected the phosphatase dead mutant Pten C124S, into *Pten*^−/−^ cells. This Pten mutant upregulated Top2a to a level similar to wild type Pten (Supplementary Fig. 3c lane 1 vs lane 4), indicating PTEN maintains TOP2A stability in a manner that is at least partially phosphatase independent. Restoration of Pten or Top2a in Pten-deficient MEFs rescued the G2 decatenation checkpoint and cells were restrained from entering mitosis (Fig. 3c). This result indicates that PTEN dependent TOP2A stabilization is sufficient for proper activation of the decatenation checkpoint. In order to compare the influence of PTEN and TOP2A on the process of decatenation, we generated HCT116 *TOP2A*^+/−^ cells with the CRISPR technique. Down regulation of TOP2A protein levels in these cells was confirmed by western blotting (Supplementary Fig. 5a lane 3 vs lane 4). Loss of one TOP2A allele resulted in an increase in the proportion of PICH bridge containing cells, and there was no significant difference in the occurrence of PICH bridges in anaphase HCT116 *PTEN*^−/−^ cells and HCT116 *TOP2A*^+/−^ cells (Fig. 3d). To further demonstrate that PTEN dependent TOP2A stabilization is essential for decatenation, we expressed ectopic PTEN (Fig. 3e, supplementary Fig. 5c lane 1 vs lane 3) or TOP2A (Fig. 3f) in PTEN deficient HCT116 cells, and both of these experiments resulted in a decrease in PICH bridge containing anaphase cells.

### Association of PTEN and TOP2A is required for regulation of the decatenation process and TOP2A levels

To explore the interaction of PTEN and TOP2A in the regulation of TOP2A levels and the decatenation process, we performed in Silico docking analysis with the structures available for these two proteins (Supplementary Fig. 4a, 4b). The structure of TOP2A, composed of an ATPase domain (29–405) and a DNA cleavage core (433–1092)^51^,^52^, was docked with the PTEN crystal structure^53^, yielding a single high probability model of the PTEN/TOP2A complex. The C-tail of TOP2A binds to the PTP domain of PTEN, including Arg47, Asp52, Lys163 and Lys164 through complimentary electrostatic interaction to within a distance of 1.7 A^°^ (Supplementary Fig. 4a, 4b). Mutations of predicted binding sites Arg47, Asp52 and Lys164 reduced the association of PTEN and TOP2A (Supplementary Fig. 4c). In addition, overexpression of R47A, D52A and K164A mutants in *PTEN*^−/−^ cells failed to upregulate TOP2A to similar level of wild-type PTEN (Supplementary Fig. 4d). In comparison with wild type PTEN, the PTEN K164A mutant did not reduce the occurrence of UFBs when overexpressed in *PTEN*^−/−^ cells (Supplementary Fig. 4e). These data indicate that association of TOP2A and PTEN plays a critical role in the regulation of TOP2A levels and the decatenation process.

### PTEN regulates stability of TOP2A in an ubiquitin dependent manner

**Figure 4:**
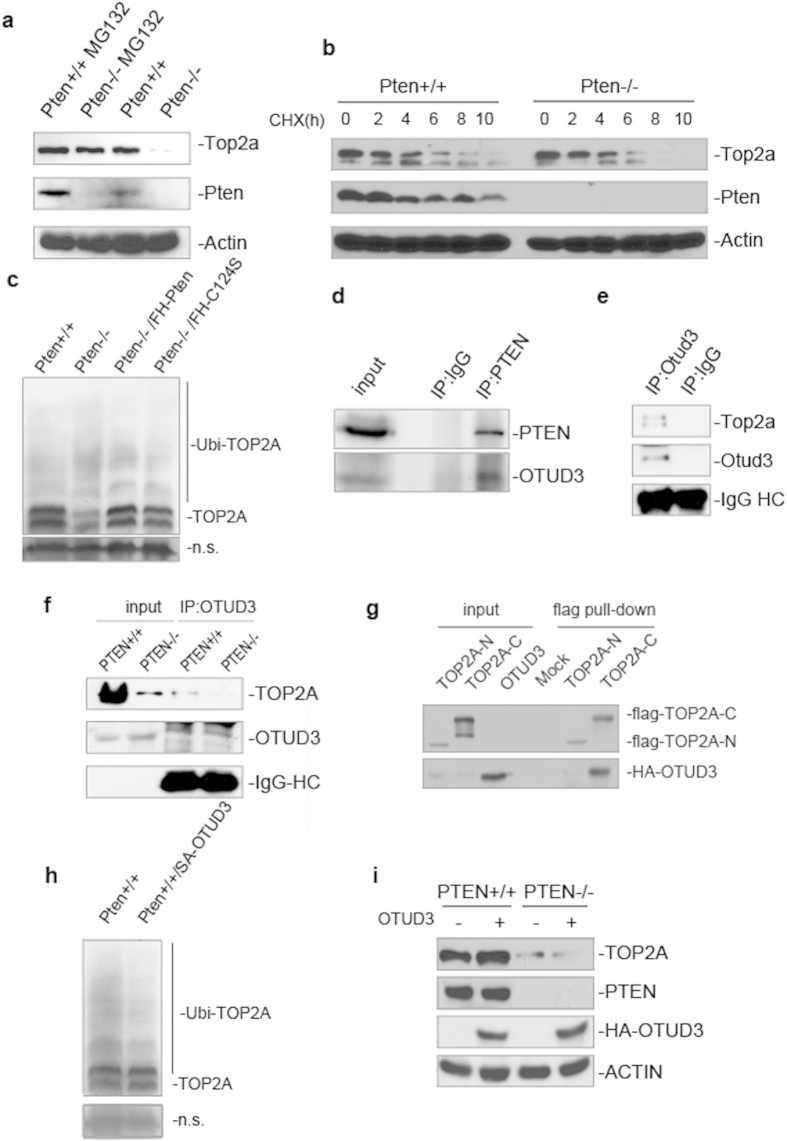
PTEN maintains TOP2A stability by promoting OTUD3 catalyzed TOP2A deubiquitination. (**a**) Western blot analysis of Top2a and Pten. MEF *Pten*^+/+^ or *Pten*^−/−^ cells were treated with MG132 prior to western blot analysis with indicated antibodies. (**b**) Western blot analysis of Top2a and Pten. MEF *Pten*^+/+^ or *Pten*^−/−^ cells were treated with MG132 prior to CHX treatment for indicated time periods followed by western blot analysis of Top2a and Pten levels. (**c**) Ubiquitination assays. HCT116 *PTEN*^+/+^ or *PTEN*^−/−^ cells, and HCT116 *PTEN*^−/−^ cells ectopically expressing Flag-tagged PTEN or PTEN C124S were transfected with His-ubiquitin and treated with MG132 prior to His-tagged pull-down and western blot analysis of ubiquitinated-TOP2A with antibody to TOP2A. Non-specific bands are shown as loading control. (**d**) Co-immunoprecipitation assays. PTEN antibody or IgG was used for immunoprecipitation in HCT116 cell extracts prior to western blotting of PTEN and OTUD3. (**e, f**) Co-immunoprecipitation assays. MEFs, HCT116 *PTEN*^+/+^ or HCT116 *PTEN*^−/−^ cell extracts were immunoprecipitated with OTUD3 antibody or mouse IgG. Western blot was then used to evaluate for presence of TOP2A and OTUD3. (**g**) OTUD3 and TOP2A *in vitro* binding assay. SA-tagged OTUD3 and flag-tagged TOP2A-N and TOP2A-C were purified and incubated together before flag pull-down. Note that the C-terminus of TOP2A is able to bind OTUD3 *in vitro*. (**h**) Ubiquitination assays. HCT116 cells were transfected with either empty vector or HA-OTUD3 together with His-ubiquitin prior to MG132 treatment. Ubiquitinated proteins were enriched with His pull-down and blotted with TOP2A antibody. Non-specific bands are shown as loading control. (**i**) Western blot analysis of TOP2A, PTEN and overexpressed HA-OTUD3. HA-tagged OTUD3 was overexpressed in HCT116 *PTEN*^+/+^ and *PTEN*^−/−^ cells before western blotting with indicated antibodies.

PTEN controls several proteins by proteasome-dependent degradation^43^,^54^,^55^. To determine whether PTEN regulates TOP2A stability in this manner, we treated MEF cells with the proteasome inhibitor MG132. After treatment with MG132, levels of Top2a did not decrease in *Pten*^−/−^ cells, arguing that Pten controls proteasome mediated degradation of Top2a (Fig. 4a lane 1 vs lane 2, lane 3 vs lane 4). To further confirm Pten promotes Top2a protein stability, we accumulated protein using MG132, then replaced this proteasome inhibitor with the translational inhibitor CHX and observed the degradation of Top2a over time. Top2a was degraded more quickly in *Pten*^−/−^ cells than in *Pten*^+/+^ cells (Fig. 4b lane 1–6 vs lane 7–12), confirming that PTEN stabilizes TOP2A protein. In order to investigate the influence of PTEN on TOP2A ubiquitination, we performed ubiquitination assays by overexpressing His-tagged ubiquitin plasmids in *PTEN*^+/+^ or *PTEN*^−/−^ cells, as well as in *PTEN*^−/−^ cells expressing ectopic PTEN or PTEN C124S followed by His pull-down. More ubiquitinated TOP2A was found in *PTEN*^−/−^ cells by evaluation with a TOP2A antibody (Fig. 4c lane 1 vs lane 2), and both exogenous PTEN and the phosphatase dead PTEN mutant suppress ubiquitination of TOP2A (Fig. 4c lane 2 vs lane 3, 4). In addition, we examined Top2a mRNA levels in MEF cells with qRT-PCR, and found mRNA levels were unchanged in both *Pten*^+/+^ and *Pten*^−/−^ MEFs (Supplementary Fig. 5b).

### PTEN maintains TOP2A stability through OTUD3

In order to determine how PTEN brings about ubiquitin mediated TOP2A stabilization and identify potential mediators, we performed S-tag PTEN pull down assays with cells treated with MG132. We identified several deubiquitinases associated with PTEN by mass spectrometry analysis (data not shown). We sought to determine which of these PTEN associated deubiquitinases associate with TOP2A, and found OTUD3 can associate with TOP2A. Co-IP assays confirmed interaction of PTEN and OTUD3 (Fig. 4d lane 3). This association of OTUD3 and TOP2A was confirmed in MEFs and HCT116 cells (Fig. 4e lane 2, 4f lane 3). Direct interaction between OTUD3 and TOP2A was confirmed by an *in vitro* binding assay (Fig. 4g lane 6). Moreover, overexpression of OTUD3 in *PTEN*^+/+^ cells down regulated ubiquitination of TOP2A (Fig. 4h lane 1 vs lane 2). To further confirm OTUD3 mediates PTEN induced TOP2A stabilization, OTUD3 was both overexpressed and knocked down in HCT116 cells. Overexpression of OTUD3 leads to upregulation of TOP2A in *PTEN*^+/+^ cells, but not in *PTEN*^−/−^ cells (Fig. 4i lane 1 vs lane 2, lane 3 vs lane 4). This indicates that OTUD3 mediated upregulation of TOP2A is PTEN dependent. Following OTUD3 knock-down with two RNAi sequences, TOP2A was decreased in one of the OTUD3 knock-down assays in HCT116 *PTEN*^+/+^ cells, but not in *PTEN*^−/−^ cells (Supplementary Fig. 5d lane 1 vs lane 3). Moreover, more UFBs were observed in OTUD3 knock-down cells (Supplementary Fig. 5e). Taken together, these results argue that PTEN promotes TOP2A stability through OTUD3 mediated deubiquitination.

## Discussion

Chromosomal instability results from defects in a network of genes regulating cell cycle checkpoints, chromosomal organization and segregation. In this study, we describe a novel PTEN function which serves to protect chromosomal stability. We demonstrate that PTEN facilitates the DNA decatenation process and protects the decatenation checkpoint. PTEN loss elicits generation of ultra-fine bridges occurring secondary to incomplete decatenation, and pseudomitosis. When treated with decatenation inhibitor, PTEN deficient cells exhibit accumulation but not reduction of mitotic cells, reflecting attenuation of the G2 phase decatenation checkpoint. PTEN interacts with the critical decatenation regulator TOP2A *in vitro* and *in vivo*, and maintains its protein stability by recruiting the deubiquitinase OTUD3. A finding of particular interest is that overexpression of OTUD3 in *PTEN*^+/+^ cells but not in *PTEN*^−/−^ cells upregulates TOP2A protein levels, indicating PTEN is required for OTUD3 function. Our study both expands knowledge of PTEN functions involved in chromosomal stabilization, and reveals a mechanism by which TOP2A protein is regulated. TOP2A is required for proper decatenation and mitosis, and this provides mechanistic insight into basic cellular function and also suggests a mechanism by which DNA damage may accumulate in the absence of PTEN during tumorigenesis.

PTEN is a tumor suppressor that regulates a wide range of cellular and physiologic events, and functions extending beyond its originally identified canonical phosphatase activity have been identified and defined^12^,^20^,^43^,^48^. We have previously reported that PTEN maintains centromere stability^20^, and PTEN C-terminal deletion in a mouse model elicits phenotypes of genomic instability such as centromeric abnormalities and fragile site instabilities^12^. Our study here provides another mechanism underlying PTEN function for maintaining genomic stability. One third of the ultra-fine bridges induced by PTEN loss are found in the centromeric region, which offers a possible explanation for PTEN related centromeric instability. A previous study by our laboratory demonstrated that loss of PTEN results in dissociation of histone H1 from chromatin and decondensation of chromatin^48^. TOP2A is required for compaction of mitotic chromosomes^56^,^26^, and decreases in TOP2A may thus also contribute to chromatin remodeling aberrations in PTEN deficient cells.

The DNA damage checkpoint also functions during G2, however the decatenation checkpoint is distinct from the DNA damage checkpoint, and the DNA damage checkpoint may remain intact in cells where the decatenation checkpoint is compromised 46,47,57. Loss of PTEN results in inhibition of Chk1, as Chk1 fails to enter the nucleus after phosphorylation mediated ubiquitination, thereby impairing the DNA damage checkpoint^45^,^58^. PTEN control of the decatenation checkpoint suggests that PTEN plays a supervisory role in G2 checkpoints and thus protects genomic integrity.

PTEN interacts with several E3 ubiquitin ligases and regulates the ubiquitin dependent protein degradation of the substrates of these enzymes^43^,^55^. Here we showed PTEN interacts with OTUD3 and influences stability of its downstream protein TOP2A, and this represents a novel mechanism by which PTEN regulates levels of its downstream targets.

In conclusion, our findings demonstrate PTEN plays an important role in protecting DNA decatenation and the G2 decatenation checkpoint. We provide novel evidence that PTEN is a regulator of TOP2A through maintenance of its protein stability. PTEN loss results in decatenation errors and decatenation checkpoint dysfunction, and restoration of PTEN or TOP2A in *PTEN*^−/−^ cells rescues these decatenation and checkpoint deficiencies. We propose that PTEN controls chromosomal segregation and protects genomic integrity by facilitating DNA decatenation.

## Materials and Methods

### Cell lines and culture

Primary *Pten*^+/+^ and *Pten*^*−/−*^ mouse embryonic fibroblasts (MEFs, <passage 6) were a gift from Dr. H. Wu at UCLA. The human CRC cell line DLD1 *PTEN*^+/+^ and DLD1 *PTEN*^−/−^ were obtained from Sigma-Aldrich (Catalog Number CLLS1004). HCT116 *PTEN*^+/+^ and HCT116 *PTEN*^−/−^ cells were a gift from Dr. Todd Waldmann at Georgetown University, and 293T cells were from the American Type Culture Collection. Cells were cultured in MEM (Gibco) supplemented with 10% FBS. The insect cell line Sf9 was obtained from Invitrogen and characterized by morphological analysis, and cultured in Grace’s insect medium (Gibco) with 10% FBS.

### Immunofluorescence

Cells were cultured on glass slides overnight and fixed in 3.7% in formaldehyde for 20 min at 4 °C, followed by permeabilization with 0.1% Triton X-100. Cells were blocked in 5% skim milk and incubated with antibodies against PTEN (Santa Cruz N19), TOP2A (MBL #M042-3), PICH (Abnova#H00054821-Do1p) or CREST (Immunovision HCT-0100) followed by incubation with Alexa Fluor 488 and/or 555 secondary antibodies (Invitrogen). Slides were then mounted with mounting solution with DAPI. Immunofluorescence images were acquired with a Nikon camera and quantitated with NIS Elements AR imaging software. For each channel, images were acquired with identical settings. To quantify anaphase bridges, more than three independent experiments were conducted. 40 ~ 100 anaphases or telophases were recorded from each slide.

### Cell synchronization cell cycle analysis

MEFs, HCT116 and DLD1 cells with a confluency of 20% ~ 30% were incubated in 2 mM thymidine for 18 h, released in fresh media for 8 h, then incubated in 2 mM thymidine for 18 h. Cells were washed and released in fresh media for harvest at indicated time points.

For ICRF-193 treatment, cells were incubated in 1 μM ICRF-193 for 2 h before fixation. For UV treatment, 40 J/m^2^ ultraviolet radiation was administrated to the cells. Cells were then returned to the incubator for 2 h before fixation. MEFs or HCT116 cells were fixed in 70% cold ethanol for 20 min, followed by blocking in 1% BSA for 20 min before incubation with an antibody against pH3 (S10) . Cells were washed and incubated in Alexa Fluor 488 goat anti-rabbit IgG (Invitrogen) for 20 min at room temperature. Cells were resuspended with PI and RNase for flow cytometry analysis using a FACScan (BD). Data were analyzed using FlowJo software (Tree Star).

### Extraction of chromatin-bound proteins and Western blotting

Extractions were performed as described by Mendez^59^. Briefly, the soluble cytoplasmic fraction was isolated by incubating cells in buffer A (10 mM HEPES at pH 7.9, 10 mM KCl, 1.5 mM MgCl2, 0.34 M sucrose, 10% glycerol) supplemented with protease inhibitors and 0.1% Triton-X for 5 min on ice followed by centrifugation. The supernatant was collected as the cytoplasmic fraction. Then the nuclear-insoluble fraction was isolated by resuspending the cell pellets in buffer B (3 mM EDTA, 0.2 M EGTA) supplemented with protease inhibitors for 30 min on ice followed by centrifugation. The supernatant was collected as the nuclear-soluble fraction, and pellets were collected as the nuclear-insoluble fraction. All samples were boiled for 5 min prior to protein quantification as described below, and subjected to western blotting.

### Metaphase spread and FISH assay

MEF cells with a confluency of 70%-80% were incubated with demecolcine (0.1 mg/ml) for 4 h before collection, and were swelled in 0.56% KCl for 10 min at 37 °C. Cells were then fixed in 1:3 acetic acid/methanol fixing buffer. Fixed cells were dropped on slides and dried before hybridization with probes. Slides were dried, and were then passed through a graded series of alcohols (70%, 90% and 100%) prior to baking at 65 °C for 15 min, followed by cooling and transfer to acetone for 10 min. After air drying, slides were incubated in 2× SSC + RNase prior to washing twice in 2× SSC and once for 5 min each. Slides were incubated in 10 mM HCl with 0.5 μg/ml pepsin and washed and dehydrated through a graded series of ethanol. To denature the DNA, slides were immersed in 70% formamide in 2−SSC at 70 °C for 2 min, and then placed in ice cold 70% ethanol for 2 min. Slides were dehydrated again prior to incubation with probes for 16 h at 37 °C in a humidified chamber. Images were then acquired.

### Western blotting and immunoprecipitation

70% ~ 80% confluent cells were washed twice before harvesting. To release chromatin bound proteins, cells were lysed in NP40 lysis buffer with 100 ug/ml DNase I (Sigma) and proteinase inhibitors for 30 min at 4 °C. Protein concentration was measured with a Bradford kit (Bio-rad #500-0205). For western blots, equal amounts of protein were boiled in gel loading buffer and loaded onto 8% or 10% SDS-PAGE gel. Blots were blocked in 5% skim milk, incubated with antibodies against TOP2A (MBL #M042-3), PTEN (Santa Cruz sc-7974), ACTIN (Protein Star KM9001), OTUD3 (antibody raised in our laboratory), p53 (Abcam D01) or HA (Protein Star KM8004). Blots were washed twice and incubated with secondary antibodies (Santa-Cruz) prior to identification of bands with chemiluminescence (ECL).

For MG132 treatment, cells were incubated with 1 μM MG132 for 8 h before harvesting for western blot analysis. For CHX treatment, cells were treated with 100 μg/ml CHX for indicated times.

For immunoprecipitation, cell lysates with 3 mg total protein were incubated with antibodies against PTEN, TOP2A or OTUD3 overnight, and were then incubated with Protein A/G agarose beads (Biochem). The beads were washed with immunoprecipitation buffer prior to western blot analysis.

### Affinity Purification of PTEN Protein Complex

Whole cell lysate was prepared from 5 × 10^7^ HCT116 *PTEN*^−/−^ cells transfected with an empty plasmid or a plasmid with S-tagged PTEN. After incubation with S-protein resin (Novogen) for 2 h, elutes were combined and concentrated for SDS-PAGE and coomassie blue staining. Visible protein bands were excised from the gel and subjected to mass spectrometric analysis.

### Plasmids and DsiRNA

A pFastBac1 expression vector (Invitrogen) was used to construct plasmids expressing TOP2A-N, TOP2A-C, or full-length PTEN in SF9 insect cells. The N-terminal truncated TOP2A includes amino acids 1–705 and C-terminal truncated TOP2A includes amino acids 668–1531. pET28a (+) plasmid was used for the PTEN N-terminal domain, PTEN C-terminal domain, or control proteins with His-tag. N-terminal PTEN included amino acids 1-189, and C-terminal PTEN included amino acids 190–403. Exogenous OTUD3 was overexpressed in an S-HA tagged vector. An S-protein-HA tag was fused to the N-terminal of exogenously expressed OTUD3^60^. Expression vectors containing FLAG-HA epitope tags were a gift from Dr. W. Gu at Columbia University. DsiRNAs were ordered from IDT with sequences GCCCGCGCUUUUAACCU and AGGCCAUGUCCCGAAGC. The CRISPR plasmids used for the construction HCT116 *TOP2A*^+/−^ cells targeting exon 4 of *TOP2A* with the sequence GTGGGTTTACGATGAAGATGT were gifts from Dr. Xi at Peking University and the procedure was carried out as previously described^61^. The CRISPR targeting sequence used for the construction of *PTEN*^+/−^ mice was CCAGACATGACAGCCATCATCAAAG in *PTEN* exon 1, and the procedure used for CRISPR/Cas-mediated genomic engineering to generate mice carrying mutations was carried out with reference to the work of Wang *et al*.^62^.

### Purification of Proteins from bacteria or SF9 cells and the Ni-NTA Binding Assay

His-PTEN, Flag-HA-TOP2A-N or Flag-HA-TOP2A-C fusion proteins were expressed in Sf9 insect cells and purified using Ni-NTA agarose. For purification of His-PTEN, Flag-HA-TOP2A-N or Flag-HA-TOP2A-C, 5 × 10^7^cells infected with corresponding virus were harvested by centrifugation. His-PTEN-N and His-PTEN-C protein were purified from BL21 E.coli cells expressing pET28a(+)-PTEN-N or pET28a(+)-PTEN-C plasmids. His proteins were then purified with a Ni-NTA system (Qiagen). Flag-HA-TOP2A-N and Flag-HA-TOP2A-C were purified with M2 anti-FLAG resin (Sigma). Resin was packed in columns, and Flag-HA-PTEN complex was eluted with 3× FLAG peptide.

### TopoisomeraseII assay

MEF cells from one 100 mm dish were pelleted in cold conditions then resuspended in 3 ~ 5 ml of TEMP buffer (10 mM Tris-HCl, pH 7.5, 1 mM EDTA, 4 mM MgCl2,). These cells were lysed in a glass homogenizer on ice. The nuclear pellets were collected by centrifugation at 1500 g for 10 min at 4 °C. Nuclear pellet were then resuspended in 30 ~ 50 ml of TEM buffer (10 mM Tris-HCl, pH 7.5, 1 mM EDTA). After addition of an equal volume of 1M NaCl, samples were left on ice for 1 h. The samples were centrifuged at 12000 g in a microcentrifuge for 15 min, and 2 μg of protein containing supernatant was used for determination of Topo II activity. Activity of Topoisomerase II was evaluated with a kit from Topogen (TG1001–1).

### qPCR analysis

MEF cells with a confluency of ~70% were harvested for mRNA extraction with TRIZOL reagent (Invitrogen). Kits for reverse transcription and Master Mix were purchased from Promega. qPCR analysis was conducted with 7500 Real-Time PCR System (Applied Biosystems). PCR amplification was performed for 40 cycles at 94 °C for 30 s, 60 °C for 30 s, and 72 °C for 30 s, with 1.0 μl of cDNA and SYBR Green Real-time PCR Master Mix. Data was collected and analyzed with SDS 2.3 software (Applied Biosystems). The expression level of TOP2A was internally normalized against that of glyceraldehyde 3-phosphate dehydrogenase (GAPDH). Each experiment was performed in triplicate and repeated three times. The PCR primers for TOP2A were: sense–ACCATTGCAGCCTGTAAATGA; antisense–GGGCGGAGCAAAATATGTTCC.

## Additional Information

**How to cite this article**: Kang, X. *et al*. PTEN stabilizes TOP2A and regulates the DNA decatenation. *Sci. Rep*. **5**, 17873; doi: 10.1038/srep17873 (2015).

## Supplementary Material

Supplementary Information
