# Supplementary material for: PTEN stabilizes TOP2A and regulates the DNA decatenation
Source: Sci Rep. 2015 Dec 10;5:17873. doi: 10.1038/srep17873 (PMC4674714; doi:10.1038/srep17873)
Supplement: Supplementary Information [file srep17873-s1.pdf]

Supplementary materials

PTEN stabilizes TOP2A and regulates the DNA decatenation

Xi Kang[1,2], Chang Song[1], Xiao Du[1], Cong Zhang[1], Yu Liu[1], Ling Liang[1], Jinxue He[2], Kristy Lamb[2], Wen H. Shen[2,*] & Yuxin Yin[1,2,3,*]

[1]Institute of Systems Biomedicine, Beijing Key Laboratory of Tumor Systems Biology, Department of Pathology, School of Basic Medical Sciences, Peking University Health Science Center, Beijing 100191, China

[2]Department of Radiation Oncology, Weill Medical College of Cornell University, New York, NY 10065, USA

[3]Peking-Tsinghua Center for Life Sciences, Beijing, 100191, China

[*]Correspondences:

Yuxin Yin, M.D., Ph.D.
University Professor and Director
Institute of Systems Biomedicine
Dean, School of Basic Medical Sciences
Peking University Health Science Center
Beijing 100191, China
Phone: (86) 10-8280-1237
Fax: (86) 10-8280-1380
E-mail: yinyuxin@hsc.pku.edu.cn

Wen H. Shen, Ph.D.
Assistant Professor
Department of Radiation Oncology
Weill Cornell Medical College
New York, NY 10021
Phone: (212) 746-1314
Fax: (212) 746-0095
E-mail: wes2007@med.cornell.edu

Figure S1

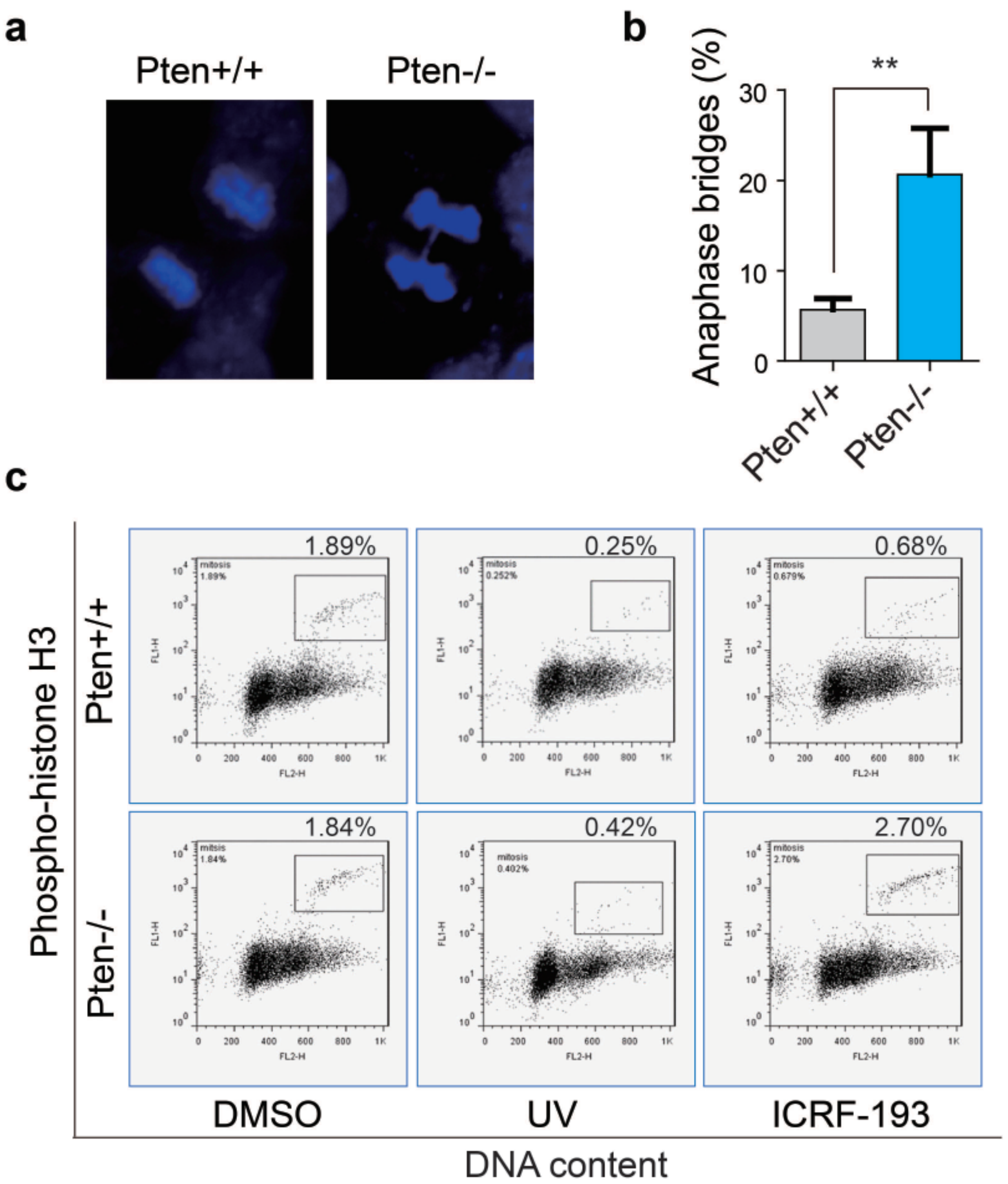
a
Pten+/+
Pten-/-
b
Anaphase bridges (%)
**
30
20
10
0
Pten+/+
Pten-/-
c
Phospho-histone H3
Pten+/+
Pten-/-
1.89%
0.25%
0.68%
1.84%
0.42%
2.70%
DMSO
UV
ICRF-193
DNA content

Supplementary Figure 1. PTEN deficiency results in anaphase bridges and decatenation checkpoint dysfunction. (**a**) DAPI bridge staining. Anaphase bridges were counted in MEF *Pten*$^{+/+}$and *Pten*$^{-/-}$ cells after staining with DAPI. (**b**) Statistical analysis of **A**. Data represent 3 independent experiments in S1A ± SEM, n=40, One-Way ANOVA, ** p<0.01. (**c**) Flow cytometry analysis of mitotic cells. Representative spot graphs of dual color flow cytometry analysis of **Figure 1g**.

Figure S2

a

```
MEVSPLQPVNENMQVNKIKKNEDAKKRLSVERIYQKKTQLEHILLRPDTYIGSVELVTQQMWVYDEDVGIN
YREVTFVPGLYKIFDEILVNAADNKQRDPKMSCIRVTIDPENNLISIWNNGKGIPVVEHKVEKMYVPALIF
GQLLTSSNYDDDEKKVTGGRNGYGAKLCNIFSTKFTVETASREYKKMFKQTWMDNMGRAGEMELKPFNGED
YTCITFQPDLSKFKMQSLDKDIVALMVRRAYDIAGSTKDVKVFLNGNKLPVKGFRSYVDMYLKDKLDETGN
SLKVIHEQVNHRWEVCLTMSEKGFQQISFVNSIATSKGGRHVDYVADQIVTKLVDVVKKKNKGGVAVKAHQ
VKNHMWIFVNALIENPTFDSQTKENMTLQPKSFGSTCQLSEKFIKAAIGCGIVESILNWVKFKAQVQLNKK
CSAVKHNRIKGIPKLDDANDAGGRNSTECTLILTEGDSAKTLAVSGLGVVGRDKYGVFPLRGKILNVREAS
HKQIMENAEINNIIKIVGLQYKKNYEDEDSLKTLRYGKIMIMTDQDQDGSHIKGLLINFIHHNWPSLLRHR
FLEEFITPIVKVSKNKQEMAFYSLPEFEEWKSSTPNHKKWKVKYYKGLGTSTSKEAKEYFADMKRHRIQFK
YSGPEDDAAISLAFSKKQIDDRKEWLTNFMEDRRQRKLLGLPEDYLYGQTTTYLTYNDFINKELILFSNSD
NERSIPSMVDGLKPGQRKVLFTCFKRNDKREVKVAQLAGSVAEMSSYHHGEMSLMMTIINLAQNFVGSNNL
NLLQPIGQFGTRLHGGKDSASPRYIFTMLSSLARLLFPPKDDHTLKFLYDDNQRVEPEWYIPIIPMVLING
AEGIGTGWSCKIPNFDVREIVNNIRRLMDGEEPLPMLPSYKNFKGTIEELAPNQYVISGEVAILNSTTIEI
SELPVRTWTQTYKEQVLEPMLNGTEKTPPLITDYREYHTDTTVKFVVKMTEEKLAEAERVGLHKVFKLQTS
LTCNSMVLFDHVGCLKKYDTVLDILRDFFELRLKYYGLRKEWLLGMLGAESAKLNNQARFILEKIDGKIII
ENKPKKELIKVLIQRGYDSDPVKAWKEAQQKVPDEEENEESDNEKETEKSDSVTDSGPTFNYLLDMPLWYL
TKEKKDELCRLRNEKEQELDTLKRKSPSDLWKEDLATFIEELEAVEAKEKQDEQVGLPGKGGKAKGKKTQM
AEVLPSPRGQRVIPRITIEMKAEAEKKNKKKIKNENTEGSPQEDGVELEGLKQRLEKKQKREPGTKTKKQT
TLAFKPIKKGKKRNPWSDSESDRSSDESNFDVPPRETEPRRAATKTKFTMDLDSDEDFSDFDEKTDDEDFV
PSDASPPKTKTSPKLSNKELKPQKSVVSDLEADDVKGSVPLSSSPPATHFPDETEITNPVPKKNVTVKKTA
AKSQSSTSTTGAKKRAAPKGTKRDPALNSGVSQKPDPAKTKNRRKRKPSTSDDSDSNFEKIVSKAVTSKKS
KGESDDFHMDFDSAVAPRAKSVRAKKPIKYLEESDEDDLF
```

b

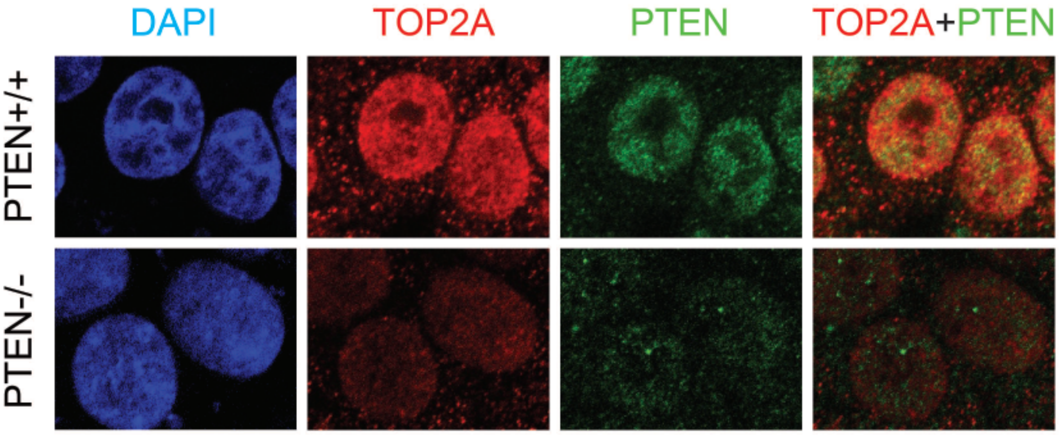

Supplementary Figure 2. PTEN associates with TOP2A. (**a**) S-tag pull down assays. Specific peptides captured with the S-tagged PTEN pull down assay in Figure2A, and sequences which are identical with TOP2A are highlighted. (**b**) Immunofluorescence analysis. Immunofluorescent imaging of fixed HCT116 *PTEN*$^{+/+}$ and *PTEN*$^{-/-}$ cells showing co-localization of TOP2A (red) and PTEN (green). The nucleus is stained with DAPI.

Figure S3

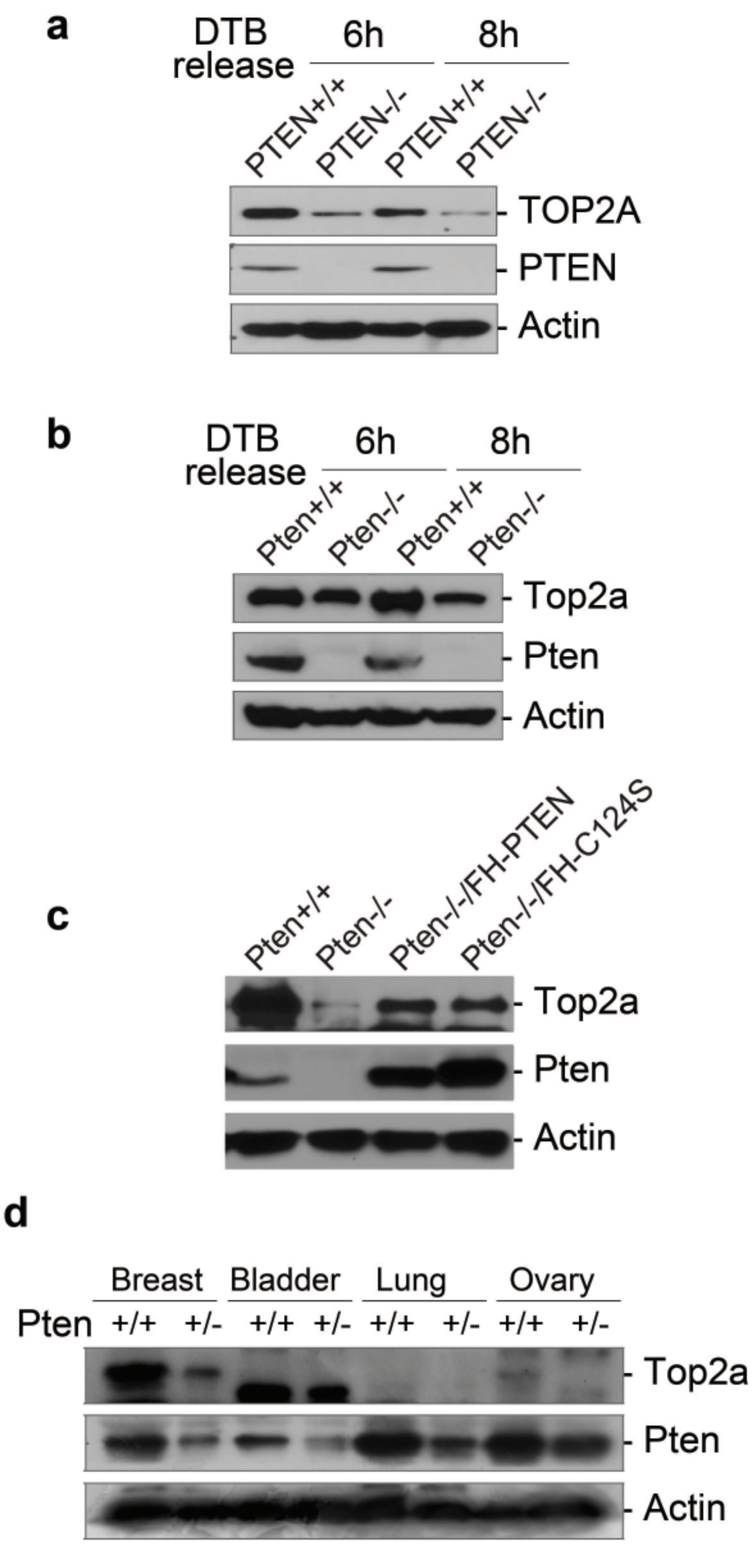

a
DTB release
6h
8h
PTEN+/+
PTEN-/-
PTEN+/+
PTEN-/-
TOP2A
PTEN
Actin
b
DTB release
6h
8h
Pten+/+
Pten-/-
Pten+/+
Pten-/-
Top2a
Pten
Actin
c
Pten+/+
Pten-/-
Pten-/-/FH-PTEN
Pten-/-/FH-C124S
Top2a
Pten
Actin
d
Breast
Bladder
Lung
Ovary
Pten
+/+
+/-
+/+
+/-
+/+
+/-
+/+
+/-
Top2a
Pten
Actin

Supplementary Figure 3. PTEN maintains protein levels of TOP2A. (**a**) Western blot analysis. HCT116 *PTEN*$^{+/+}$ and *PTEN*$^{-/-}$ cells were synchronized with double thymidine block and released for 6h or 8h prior to evaluation of TOP2A and PTEN protein levels. (**b**) Western blot analysis. MEF *Pten*$^{+/+}$ and *Pten*$^{-/-}$ cells were synchronized with double thymidine block and released for 6h and 8h prior to western blotting with indicated antibodies. (**c**) Western blot analysis. Flag-HA tagged wild type Pten or C124S mutant were overexpressed in *Pten*$^{-/-}$ MEFs. Western blotting show the protein levels of Top2a and Pten. (**d**) Western blot analysis. Tissues from wild type and *Pten*$^{+/-}$ mice were analyzed for Top2a and Pten protein levels with western blotting.

Figure S4

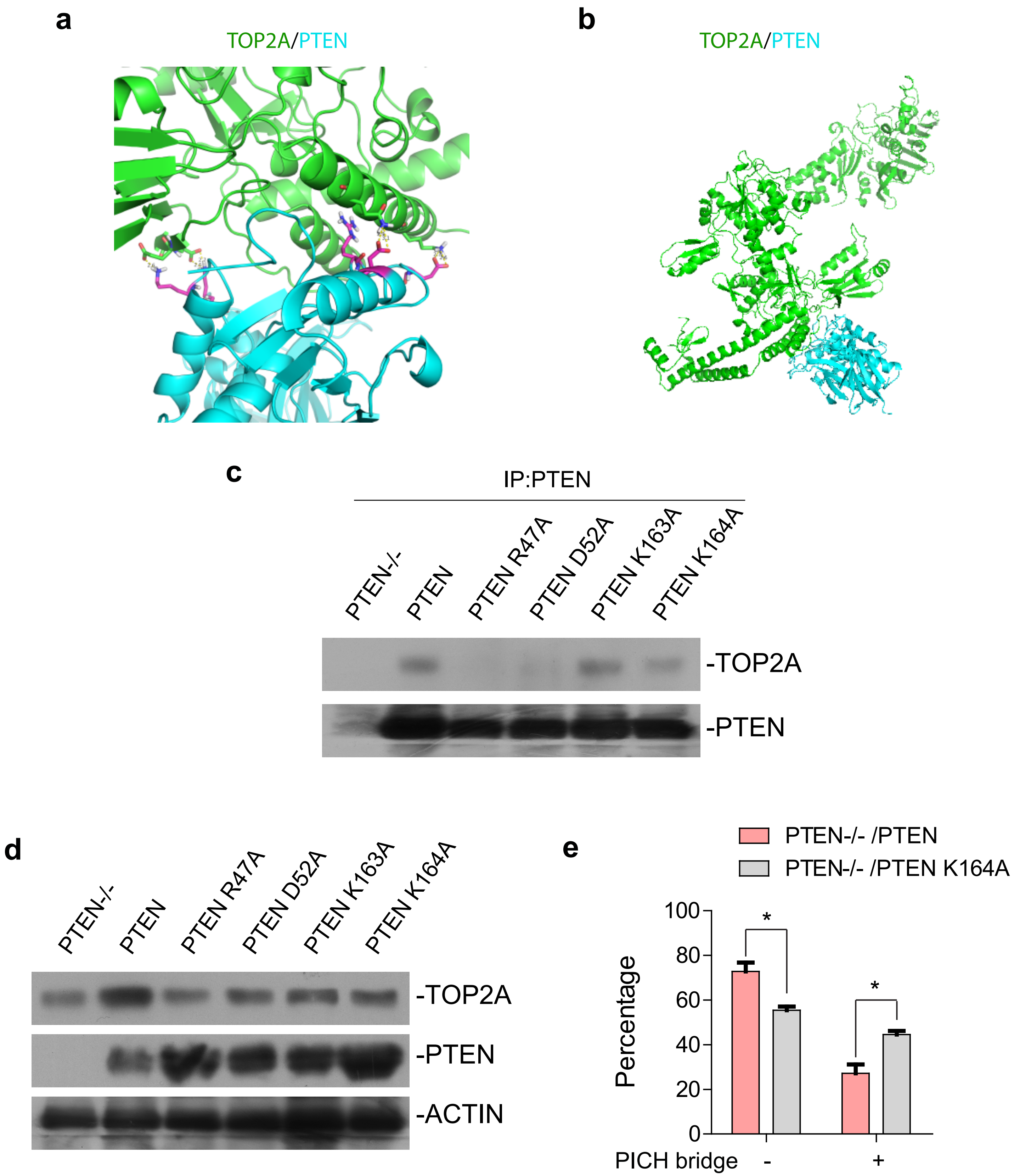

a
TOP2A/PTEN
b
TOP2A/PTEN
c
IP:PTEN
PTEN-/-
PTEN
PTEN R47A
PTEN D52A
PTEN K163A
PTEN K164A
-TOP2A
-PTEN
d
PTEN-/-
PTEN
PTEN R47A
PTEN D52A
PTEN K163A
PTEN K164A
-TOP2A
-PTEN
-ACTIN
e
PTEN-/- /PTEN
PTEN-/- /PTEN K164A
Percentage
100
80
60
40
20
0
*
*
PICH bridge
-
+

Supplementary Figure 4. Association of PTEN and TOP2A is critical for maintaining TOP2A protein levels and regulation of the decatenation process. (**a**) Illustration of in Silico docking analysis of the predicted binding sites in the PTEN/TOP2A complex to within a distance of 1.7 A°. PTEN is represented by cyan and TOP2A by green. (**b**) Overview of the in Silico docking analysis predicted PTEN/TOP2A complex. PTEN is represented by cyan and TOP2A by green. (**c**) Co-immunoprecipitation analysis. FH-tagged wild-type PTEN or PTEN mutants were overexpressed in HCT116 $PTEN^{-/-}$ cells. Western blotting shows TOP2A and PTEN after co-immunoprecipitation with the PTEN antibody in HCT116 cell lysates. (**d**) Western blot analysis of TOP2A and PTEN. FH-tagged wild-type PTEN or PTEN mutants were overexpressed in HCT116 $PTEN^{-/-}$ cells. (**e**) Statistical analysis of PICH bridge positive cells. Anaphase HCT116 $PTEN^{-/-}$ cells overexpressing wild type PTEN and PTEN K164A mutant were counted and grouped based on presence or absence of PICH bridges. Quantification data of percentage of cells with or without PICH bridges represent 3 independent experiments ± SEM. n=40, One-Way ANOVA, * $p<0.05$.

Figure S5

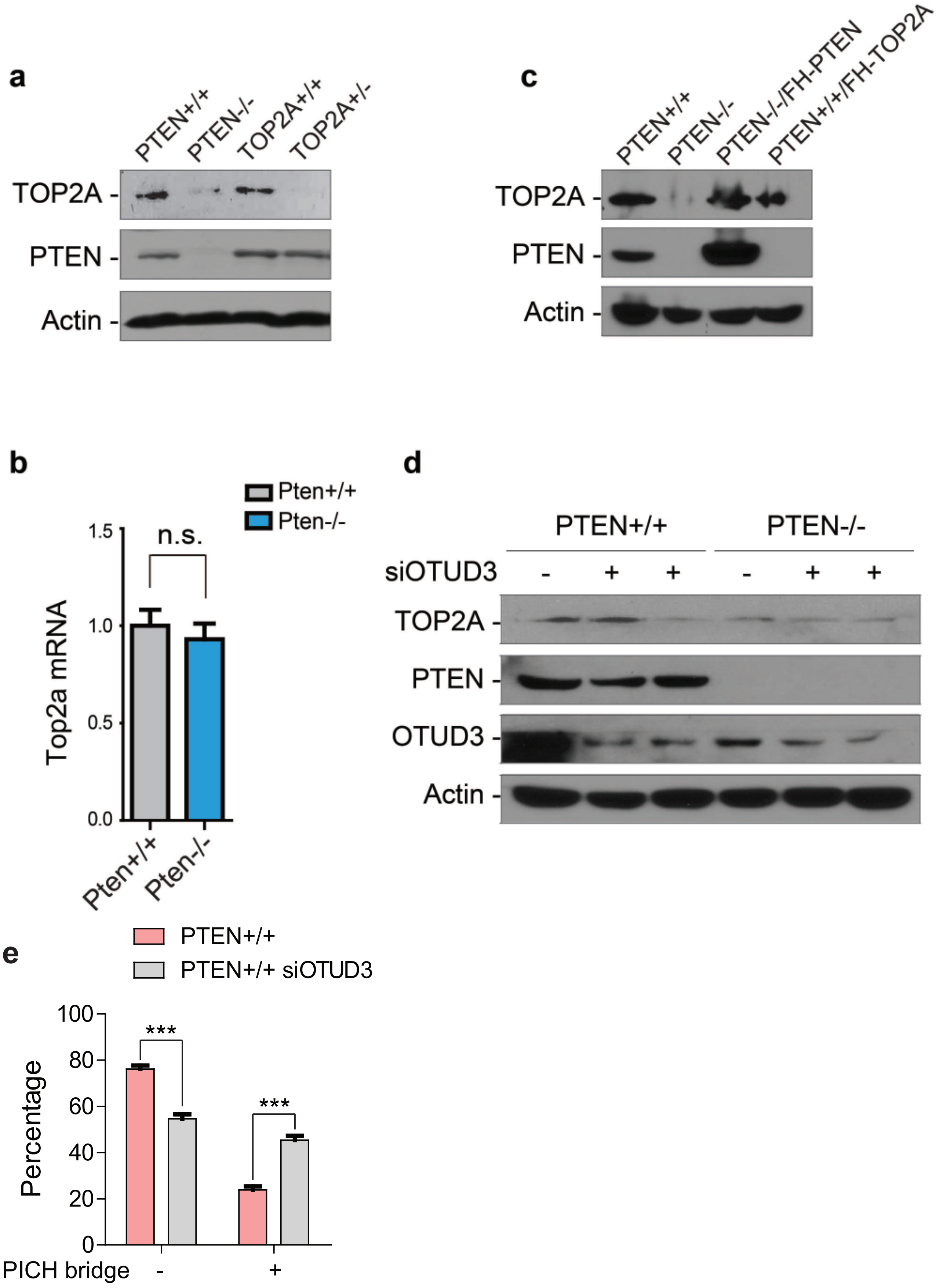

a
PTEN+/+
PTEN-/-
TOP2A+/+
TOP2A+/-
TOP2A -
PTEN -
Actin -
c
PTEN+/+
PTEN-/-
PTEN-/-/FH-PTEN
PTEN+/+/FH-TOP2A
TOP2A -
PTEN -
Actin -
b
Pten+/+
Pten-/-
n.s.
Top2a mRNA
1.5
1.0
0.5
0.0
Pten+/+
Pten-/-
d
PTEN+/+
PTEN-/-
siOTUD3
- + + - + +
TOP2A -
PTEN -
OTUD3 -
Actin -
e
PTEN+/+
PTEN+/+ siOTUD3
100
80
60
40
20
0
Percentage
***
***
PICH bridge
-
+

Supplementary Figure 5. PTEN and OTUD3 orchestrate TOP2A stability. (**a**) Western blot analysis showing levels of TOP2A and PTEN in HCT116 *PTEN*$^{+/+}$, HCT116 *PTEN*$^{-/-}$, HCT116 *TOP2A*$^{+/+}$ and HCT116 *TOP2A*$^{+/-}$ cells. (**b**) qRT-PCR analysis. Real-time PCR analysis of the transcription levels of Top2a mRNA in *Pten*$^{+/+}$ and *Pten*$^{-/-}$ cells. (**c**) Western blot analysis. Flag-tagged PTEN or TOP2A was expressed and identified in HCT116 *PTEN*$^{-/-}$ cells with TOP2A and PTEN antibodies. (**d**) Western blot analysis. Two sets of DsiRNA targeting OTUD3 were transfected into HCT116 *PTEN*$^{+/+}$ and *PTEN*$^{-/-}$ cells, and western blotting shows the expression levels of TOP2A, OTUD3 and PTEN. (**e**) Statistical analysis of PICH bridge positive cells. Anaphase HCT116 *PTEN*$^{+/+}$ cells with or without transfection of OTUD3 siRNA were counted and grouped based on presence or absence of PICH bridges. Quantification data of percentage of cells with or without PICH bridges represent 3 independent experiments ± SEM. n=40, One-Way ANOVA, *** $p<0.001$.
